# Predicting [^177^Lu]Lu-HA-DOTATATE kidney and tumor accumulation based on [^68^Ga]Ga-HA-DOTATATE diagnostic imaging using semi-physiological population pharmacokinetic modeling

**DOI:** 10.1186/s40658-023-00565-4

**Published:** 2023-08-24

**Authors:** Hinke Siebinga, Berlinda J. de Wit-van der Veen, Jos H. Beijnen, Marcel P. M. Stokkel, Thomas P. C. Dorlo, Alwin D. R. Huitema, Jeroen J. M. A. Hendrikx

**Affiliations:** 1 Department of Pharmacy and Pharmacology, The Netherlands Cancer Institute, Plesmanlaan 121, 1066 CX Amsterdam, The Netherlands; 2 Department of Nuclear Medicine, The Netherlands Cancer Institute, Amsterdam, The Netherlands; 3 Graduate School of Life Sciences, Utrecht University, Utrecht, The Netherlands; 4 Department of Pharmacy, Uppsala University, Uppsala, Sweden; 5 Department of Clinical Pharmacy, University Medical Center Utrecht, Utrecht University, Utrecht, The Netherlands; 6 Department of Pharmacology, Princess Máxima Center for Pediatric Oncology, Utrecht, The Netherlands

**Keywords:** [^68^Ga]Ga-HA-DOTATATE, [^177^Lu]Lu-HA-DOTATATE, Theranostics, PRRT, NLMEM, Uptake prediction, Precision medicine

## Abstract

**Background:**

Prediction of [^177^Lu]Lu-HA-DOTATATE kidney and tumor uptake based on diagnostic [^68^Ga]Ga-HA-DOTATATE imaging would be a crucial step for precision dosing of [^177^Lu]Lu-HA-DOTATATE. In this study, the population pharmacokinetic (PK) differences between [^177^Lu]Lu-HA-DOTATATE and [^68^Ga]Ga-HA-DOTATATE were assessed and subsequently [^177^Lu]Lu-HA-DOTATATE was predicted based on [^68^Ga]Ga-HA-DOTATATE imaging.

**Methods:**

A semi-physiological nonlinear mixed-effects model was developed for [^68^Ga]Ga-HA-DOTATATE and [^177^Lu]Lu-HA-DOTATATE, including six compartments (representing blood, spleen, kidney, tumor lesions, other somatostatin receptor expressing organs and a lumped rest compartment). Model parameters were fixed based on a previously developed physiologically based pharmacokinetic model for [^68^Ga]Ga-HA-DOTATATE. For [^177^Lu]Lu-HA-DOTATATE, PK parameters were based on literature values or estimated based on scan data (four time points post-injection) from nine patients. Finally, individual [^177^Lu]Lu-HA-DOTATATE uptake into tumors and kidneys was predicted based on individual [^68^Ga]Ga-HA-DOTATATE scan data using Bayesian estimates. Predictions were evaluated compared to observed data using a relative prediction error (RPE) for both area under the curve (AUC) and absorbed dose. Lastly, to assess the predictive value of diagnostic imaging to predict therapeutic exposure, individual prediction RPEs (using Bayesian estimation) were compared to those from population predictions (using the population model).

**Results:**

Population uptake rate parameters for spleen, kidney and tumors differed by a 0.29-fold (15% relative standard error (RSE)), 0.49-fold (15% RSE) and 1.43-fold (14% RSE), respectively, for [^177^Lu]Lu-HA-DOTATATE compared to [^68^Ga]Ga-HA-DOTATATE. Model predictions adequately described observed data in kidney and tumors for both peptides (based on visual inspection of goodness-of-fit plots). Individual predictions of tumor uptake were better (RPE AUC –40 to 28%) compared to kidney predictions (RPE AUC –53 to 41%). Absorbed dose predictions were less predictive for both tumor and kidneys (RPE tumor and kidney –51 to 44% and –58 to 82%, respectively). For most patients, [^177^Lu]Lu-HA-DOTATATE tumor accumulation predictions based on individual PK parameters estimated from diagnostic imaging outperformed predictions based on population parameters.

**Conclusion:**

Our semi-physiological PK model indicated clear differences in PK parameters for [^68^Ga]Ga-HA-DOTATATE and [^177^Lu]Lu-HA-DOTATATE. Diagnostic images provided additional information to individually predict [^177^Lu]Lu-HA-DOTATATE tumor uptake compared to using a population approach. In addition, individual predictions indicated that many aspects, apart from PK differences, play a part in predicting [^177^Lu]Lu-HA-DOTATATE distribution.

**Supplementary Information:**

The online version contains supplementary material available at 10.1186/s40658-023-00565-4.

## Introduction

Somatostatin analogue (SSA)-based peptide receptor radionuclide therapy (PRRT) is increasingly used for the treatment of metastasized well-differentiated neuroendocrine tumors (NETs) [1]. As the outcome after PRRT varies among patients, selection of those suitable for therapy is guided by SSA-based molecular imaging to assess tumor load and expression of somatostatin receptors (SSTR) for individual lesions [2]. This theranostic approach has the benefit that pre-therapeutic imaging may predict treatment response and lead to more effective personalized treatment dosages [3, 4]. Interestingly, in case of NET, both organ uptake and tumor targeting have shown quite profound differences between diagnostic and peri-therapeutic imaging in the clinical setting [5–10]. As PRRT evolves toward a more dosimetry-driven treatment [11–14], these differences will become relevant and identification of physiological or pharmacokinetic (PK) factors that influence accumulation profiles are gaining importance.

Lutetium-177 ([^177^Lu]Lu-)DOTATATE shows a high inter-patient variability for both organ and tumor uptake [15] and this variability is an important rationale to pursue future personalized treatment planning based on (expected) absorbed doses. To introduce image-guided individualized dosing, the applicability of diagnostic single-time-point Gallium-68 ([^68^Ga]Ga-)DOTATATE imaging to predict the [^177^Lu]Lu-DOTATATE accumulation profiles needs to be studied. Direct extrapolation of diagnostic uptake profiles is not straightforward, since factors such as image noise, patients’ hydration status and timing of imaging post-injection all differ and are known to impact visualization of the distribution [16]. As was suggested by Velikyan et al. [17], kinetic modeling could be used to enhance extrapolation of [^68^Ga]Ga-DOTATATE early imaging time points to predict absorbed [^177^Lu]Lu-DOTATATE doses to tumors and organs.

Population PK models are a suitable approach to investigate PK parameters of both radiopharmaceuticals and their variability within a population [18, 19]. Such population PK modeling approaches are the golden standard in drug development and research of non-radiopharmaceuticals and are a proven tool to handle sparse data sampling, which is an advantage when applying this approach to nuclear imaging data [20]. A population PK model describes population-based radioactivity–time profiles (or time–activity curves (TACs)), which, combined with individual data, will give insights in how a specific patient differs from the population estimate. We hypothesize that the individual difference from these population estimates is rather similar for [^68^Ga]Ga- and [^177^Lu]Lu-DOTATATE and is mainly driven by an individual’s physiology (e.g., SSTR expression and tumor physiology). Therefore, relevant physiological aspects need to be included in the developed population PK models (i.e., using a semi-physiological approach). Still, in this case, a physiologically based pharmacokinetic (PBPK) approach was not preferable, since insights in population trends were required to individually estimate deviation from the typical population. Considering that radiopharmaceutical-related variables (e.g., injected activity and/or peptide amounts, clearance and receptor-mediated uptake) are already included in a population model, [^68^Ga]Ga-DOTATATE scan observations could be used to predict individual PK parameters of [^177^Lu]Lu-DOTATATE.

As a first step toward population PK-informed predictions, actual differences in population PK parameters between both theranostic agents have to be determined (i.e., ^68^Ga- and ^177^Lu-labeled high-affinity DOTATATE (HA-DOTATATE) in our hospital [21]). Hence, the initial aim of this study was to assess population PK differences between [^68^Ga]Ga-HA-DOTATATE and [^177^Lu]Lu-HA-DOTATATE using a semi-physiological population PK modeling approach. Subsequently, these models are then used to individually predict [^177^Lu]Lu-HA-DOTATATE kidney (organ at risk) and tumor (target tissue) uptake based on single-time-point imaging with [^68^Ga]Ga-HA-DOTATATE.

## Methods

### Patient population and imaging data

This study included data from ten patients with NETs who had received [^68^Ga]Ga-HA-DOTATATE PET/CT within six months prior to [^177^Lu]Lu-HA-DOTATATE treatment [22]. The study was approved by the Institutional Review Board of the Netherlands Cancer Institute (IRBd21187) and informed consent was obtained via institutional procedures from all individual participants included in the study.

Diagnostic PET/CT imaging was performed at ~45 min post-injection of ~100 MBq [^68^Ga]Ga-HA-DOTATATE. All patients received ~7.4 GBq [^177^Lu]Lu-HA-DOTATATE followed by post-administration imaging, including planar scintigraphy at 0.5, 4, 24 and 72 h post-injection and SPECT/CT at 24 h post-injection. [^68^Ga]Ga-HA-DOTATATE and [^177^Lu]Lu-HA-DOTATATE were prepared in house according to previously described methods [23, 24]. Patient selection, data acquisition and data analysis were described previously [22]. Individual accumulation data for spleen, kidneys and tumor lesions were available, whereas no blood samples were drawn. For tumors, only lesions with a diameter >2 cm and a maximum of five segmented lesions of which two per organ system per patient (i.e., target tumors) were included for model development. To exclude potential treatment effects on whole-body distribution, only the first cycle [^177^Lu]Lu-HA-DOTATATE was included for model development in this study. All tumor volumes were determined based on PET/CT imaging using IntelliSpace Portal (Philips Healthcare, The Netherlands) with a semi-automatic threshold segmentation method of 50% SUV_max_ [25]. Decay corrected peptide concentrations (µg/L) were used for model development and evaluation, which were calculated based on the measured radioactivity per volume and the administered specific activities (MBq/µg) (using total administered peptide amounts).

### Model development

**Fig. 1:**
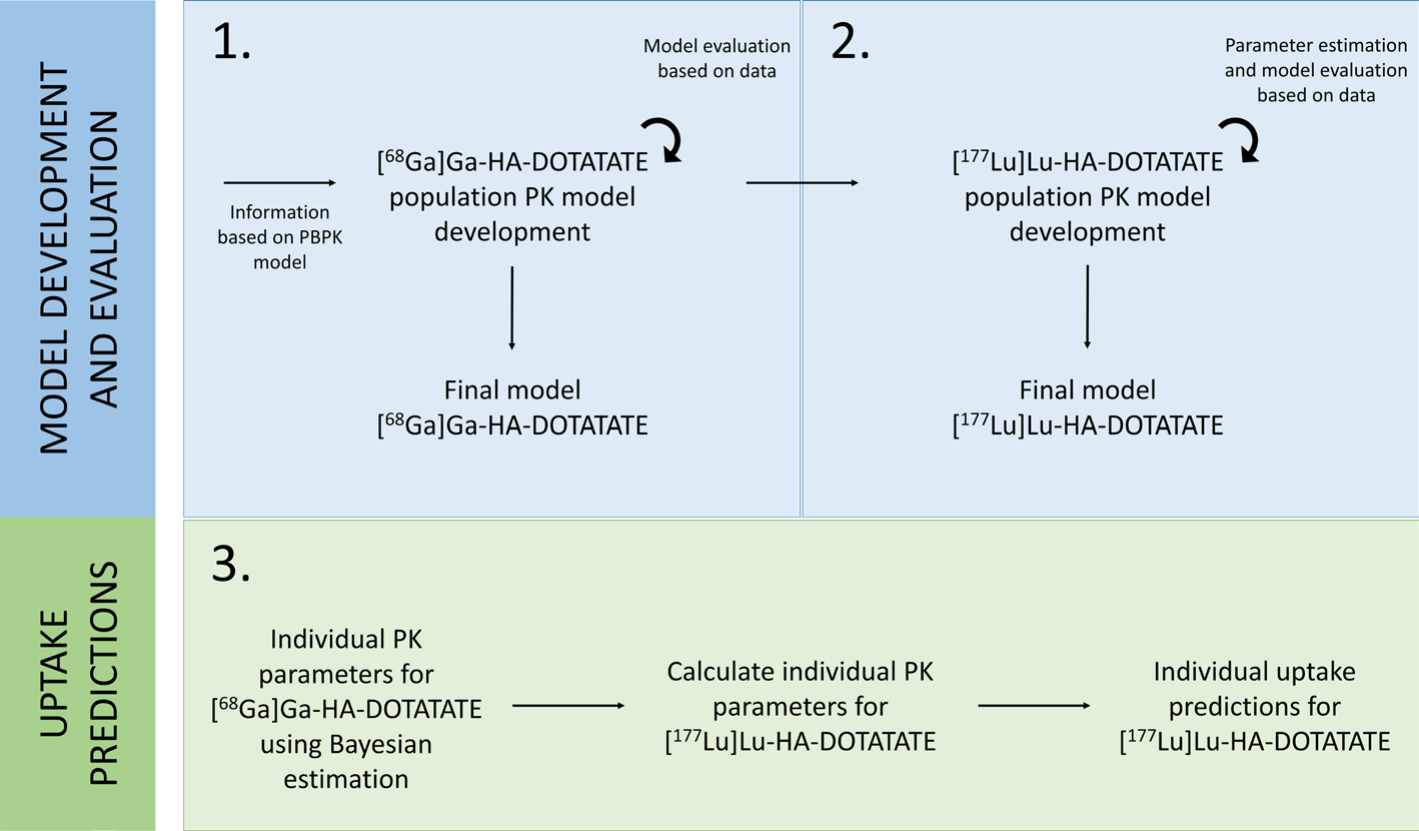
Overview of the study workflow including model development and evaluation for [^68^Ga]Ga-HA-DOTATATE and [^177^Lu]Lu-HA-DOTATATE and individual uptake predictions for [^177^Lu]Lu-HA-DOTATATE

In order to describe and compare differences in (population) PK between [^68^Ga]Ga-HA-DOTATATE and [^177^Lu]Lu-HA-DOTATATE, two semi-physiological nonlinear mixed-effects models (NLMEMs) with a similar model structure were developed. Using this approach, differences in population PK parameters between both radiopharmaceuticals were determined. An overview of the workflow is provided in Fig. 1. First, a population PK model of [^68^Ga]Ga-HA-DOTATATE was developed fully informed by a previously developed PBPK model [26]. Subsequently, the model structure was applied to [^177^Lu]Lu-HA-DOTATATE and parameters describing differences between both radiopharmaceuticals were estimated. Finally, both developed models were used to predict [^177^Lu]Lu-HA-DOTATATE PK based on [^68^Ga]Ga-HA-DOTATATE observations.

#### Semi-physiological PK model [^68^Ga]Ga-HA-DOTATATE

**Fig. 2:**
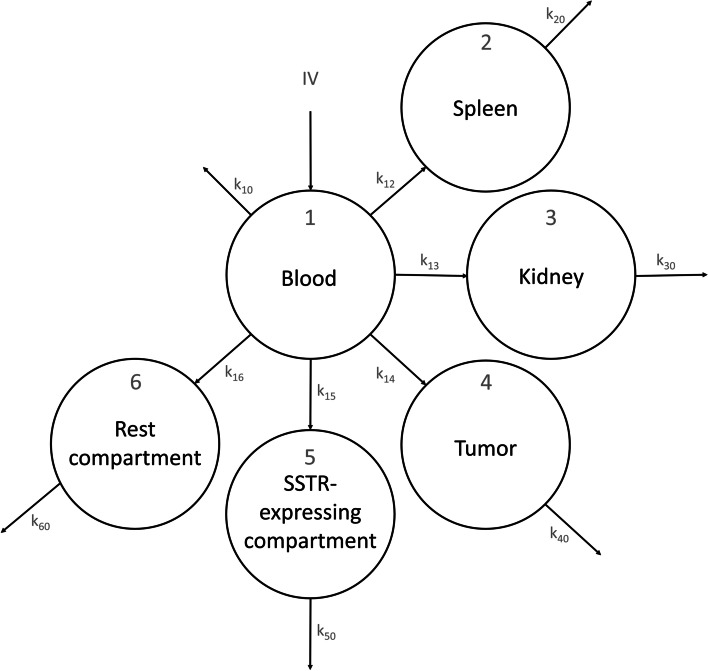
Overview of the model six-compartment structure for both the [^68^Ga]Ga-HA-DOTATATE and [^177^Lu]Lu-HA-DOTATATE models

The first step of our approach was to develop a semi-physiological model for [^68^Ga]Ga-HA-DOTATATE. Structural model development was informed based on a previously published PBPK model [26], where compartments were lumped based on their SSTR-expressing nature. This resulted in a six-compartment structural model, where compartments one to six represented the blood compartment, spleen, kidney, tumor lesions, other SSTR-expressing organs and a lumped rest compartment, respectively. Compartment five consisted of the lungs, pancreas, stomach, thyroid and liver. The full model structure is presented in Fig. 2. Structural model parameters were based on estimates from a previously published PBPK model [26]. Organ volumes were fixed for all patients and derived from the ICRP Publication 89 adult human model [27]. The tumor compartment volume was based on individual measured tumor volumes and consisted of the sum of volumes of all segmented tumors (based on [22]).

Uptake into organs and tumor occurs by internalization of the radiopharmaceutical after SSTR receptor binding [28], which was included for all SSTR-expressing compartments (two, three, four and five). Degradation from all compartments was described by a degradation rate constant (*k*_20_, *k*_30_, *k*_40_, *k*_50_ and *k*_60_) and was fixed to 0.01 h^−1^ based on previously published PBPK models [26, 29, 30]. Uptake was modeled using a maximal binding capacity (*B*_MAX_) model (Eq. 1), where occupancy of the receptor was taken into account and free binding sites decreased with uptake of each molecule into that compartment (i.e., the amount of radiopharmaceutical that was already internalized into the compartment). *B*_MAX_ amounts were calculated based on the compartment volume, thus, for compartment four, larger tumors had a higher maximal SSTR binding capacity. In addition, fractions bound to plasma proteins were taken into account and only unbound molecules were available for uptake into compartments.

1 $$\frac{\mathrm{d}A}{\mathrm{d}t}={k}_{\mathrm{in}}* {fu}*{A}_{\mathrm{blood}}*\left(1- \frac{A}{{B}_{\mathrm{MAX}}}\right)- {k}_{\mathrm{out}}* A$$

where *k*_*in*_ and *k*_out_ represent the rate constants into a specific compartment, *fu* is the unbound fraction of the radiopharmaceutical in the blood compartment, *A*_blood_ and *A* represent the compound amounts in the blood and target compartment, respectively, and *B*_MAX_ is the maximum binding capacity in the target compartment (i.e., SSTR receptor expression).

The stochastic or statistical model accounted for inter-individual variability (IIV) and residual unexplained variability. For [^68^Ga]Ga-HA-DOTATATE, IIV was added to uptake rate parameters. IIV was fixed based on assumed population variability, using information from ranges included in the previously developed PBPK model, assumed variability in uptake in organs and tumors based on clinical observations and estimated IIV in previously published population PK models for radiolabeled SSAs [26, 31, 32]. IIV was modeled using an exponential distribution according to Eq. 2, while a proportional error model described the residual error according to Eq. 3.

2 $${P}_{i}={P}_{\mathrm{pop}}*{e}^{{\eta }_{i}}$$

3 $${C}_{\mathrm{obs}, ij}={C}_{\mathrm{pred}, ij}*(1+ {\varepsilon }_{p, ij})$$

where* P*_*i*_ represents the individual PK parameter, *P*_pop_ represents the population PK parameter, and *η*_*i*_ represents the IIV effect for the *i*th individual with mean 0 and variance *ω*^2^. *C*_obs_ represents the observed concentration and *C*_pred_ the predicted concentration for the *i*th individual and the *j*th measurement, and *ε*_*p*_ the proportional error with mean 0 and variance *σ*^2^ for the *i*th individual and the *j*th measurement.

Factors known to affect PK parameters were added to the model a priori, since model development was based on fixed parameters rather than estimated based on data. Radiopharmaceutical distribution in NET patients might be impacted by a tumor sink effect, which reflects a reduced uptake in healthy tissues due to a high burden of disease [33]. Therefore, the tumor sink effect was included in the model following Eq. 4, where the extent of this effect was based on previous PBPK simulations [26]. In addition, a larger tumor volume is expected to increase tumor uptake, as was also shown for [^68^Ga]Ga-PSMA-11 [34]. Therefore, individual tumor burden was added as a structural effect to uptake into tumor lesions (*k*_14_), using a power equation (see Eq. 5), so that patients with a high tumor volume will have an increased tumor uptake compared to the typical population.

4 $${k}_{12, \mathrm{cov}}={k}_{12, \mathrm{pop}}*{e}^{0.4*(-{V}_{\mathrm{tumor total}, i})}$$

5 $${k}_{14, \mathrm{cov}}={k}_{14, \mathrm{pop}}* {(\frac{{V}_{\mathrm{tumor cmt},i }}{{V}_{\mathrm{tumor cmt},\mathrm{ median} }})}^{{eff}}$$

where *k*_*12,*pop_ and *k*_*14*,pop_ represent the population uptake rates for spleen (compartment two) and tumor (compartment four), respectively, *V*_tumor total, *i*_ represents an individual’s total tumor volume, *V*_tumor cmt, *i*_ represents an individual’s tumor volume of all segmented tumors (representing compartment 4), *V*_tumor cmt, median_ was the median tumor volume of the tumor compartment and *eff *represents the structural effect of tumor volume on *k*_14_.

A bottom-up approach was used for [^68^Ga]Ga-HA-DOTATATE model development and patient imaging data were only used for model evaluation. All model parameters were fixed, since our single-time-point data were limited to estimate PK parameters, and most parameters were based on the previously developed PBPK model [26]. The fraction of [^68^Ga]Ga-HA-DOTATATE bound to plasma proteins, renal excretion and structural effect of tumor volume on *k*_14_ (see Eq. 5) were fixed to 0.69 and 0.25 h^−1^ and 1, respectively [26]. Eventual concentration over time predictions based on the semi-physiological population model for [^68^Ga]Ga-HA-DOTATATE were visually evaluated using observed concentration–time data.

#### [^177^Lu]Lu-HA-DOTATATE parameter estimation and model evaluation

The second step was to develop the [^177^Lu]Lu-HA-DOTATATE model, and for this all initial model parameters were based on the final model for [^68^Ga]Ga-HA-DOTATATE. Most parameters were assumed similar for both agents. Renal excretion is known to be increased for [^177^Lu]Lu-HA-DOTATATE compared to [^68^Ga]Ga-HA-DOTATATE and, therefore, *k*_10_ was fixed to 0.575 h^−1^ based on a clearance value of 2.3 L/h as was estimated by Puszkiel et al. [31]. Administration of an amino acid solution during therapy compared to no kidney protection during diagnostic imaging was taken into account, since this used renal excretion rate was estimated for patients that received an amino acid solution during administration of [^177^Lu]Lu-DOTATATE. Fraction unbound in plasma was fixed to 0.57 [35]. Uptake into spleen, kidney and tumor as well as degradation from tumor were expected to differ for [^177^Lu]Lu-HA-DOTATATE; thus, the PK parameters for *k*_12,_ *k*_13_, *k*_14,_ *k*_40_ and the structural effect of tumor volume on *k*_14_ (see Eq. 5) were sequentially estimated using patient imaging data (on four time points post-injection). PK parameters for [^177^Lu]Lu-HA-DOTATATE were estimated as fold differences compared to [^68^Ga]Ga-HA-DOTATATE parameters, to enable easy comparison of final population PK parameters between both theranostic agents. Kidney data from each first imaging time point (at ~1 h post-injection) were excluded for parameter estimation, because these observations were assumed to mainly represent urine activity rather than intracellular uptake. IIV for [^177^Lu]Lu-HA-DOTATATE was added to receptor expressions rather than *k*_in_ values (as was the case for [^68^Ga]Ga-HA-DOTATATE). The rationale that receptor expressions were assumed to describe the inter-individual uptake differences was based on the differences in administered peptide amounts between the radiopharmaceuticals. For [^68^Ga]Ga-HA-DOTATATE, a very low amount of peptide is administered and receptors are not close to full occupancy, while for [^177^Lu]Lu-HA-DOTATATE (with an almost 30-fold higher administered peptide amount) uptake is mainly reliant on receptor density. A low impact of receptor expression on total uptake for [^68^Ga]Ga-HA-DOTATATE was also shown by the sensitivity analyses in our previously published PBPK models [26, 36]. Final model performance for [^177^Lu]Lu-HA-DOTATATE was evaluated based on visual inspection of predictions compared to observations and clinical plausibility of estimated parameter values was assessed.

### Individual [^177^Lu]Lu-HA-DOTATATE predictions using Bayesian estimation

The third and last step consisted of individual [^177^Lu]Lu-HA-DOTATATE uptake prediction informed by diagnostic imaging. Individual PK parameters for [^68^Ga]Ga-HA-DOTATATE were generated using Maximum a Posteriori Bayesian estimation (POSTHOC option of NONMEM, about which more information is available elsewhere [37]). Subsequently, these Bayesian estimates were used to predict individual parameters for [^177^Lu]Lu-HA-DOTATATE based on the established differences between PK parameters of both radiopharmaceuticals. The predicted individual [^177^Lu]Lu-HA-DOTATATE PK parameters, combined with individual administered dosing and covariate information, were then used to predict [^177^Lu]Lu-HA-DOTATATE concentration–time curves for all compartments.

Absorbed doses for kidney and tumor were determined to evaluate the predicted energy deposited in the irradiated tissue. For this, time-integrated activity (TIA) (MBq*h) was determined using calculated AUCs (µg*h/L) in kidney and tumors, organ and tumor volumes (L) and injected specific activity (MBq/µg). The TIA was extrapolated to infinite time based on the calculated excretion rate per individual. After this, absorbed doses were calculated following the Medical Internal Radiation Dose (MIRD) formalism [38]. Organ weights that were used for absorbed dose calculations were based on the ICRP Publication 89 adult male human model and corresponding S values were derived from IDAC-Dose 2.1 [27, 39]. For tumors, individual tumor volumes and a tissue density of 1.05 g/cm^3^ were used to determine the corresponding S value.

#### Evaluation of predictions

Predictive performance was evaluated by determining the relative prediction error (RPE) of area under the curves (AUCs) (time interval from 0 to time of the last measurement) for kidney and tumor, where predicted AUCs were compared to observed AUCs (based on four post-administration images per patient) following Eq. 6.

6 $${RPE}= \frac{{{AUC}}_{{pred}}- {{AUC}}_{{obs}}}{{{AUC}}_{{obs}}}*100\%$$

where *AUC*_*pred*_ and *AUC*_*obs*_ represent the predicted and observed AUC, respectively.

Predictions for [^177^Lu]Lu-HA-DOTATATE PK based on Bayesian estimates were compared to predictions based on the typical values in the population (i.e., the final population PK parameters, not taking [^68^Ga]Ga-HA-DOTATATE observations into account). For this, RPEs of both approaches were compared and a lower RPE was determined a better prediction.

### Software

Model development was performed in NONMEM (version 7.5; ICON Development Solutions, Ellicott City, MD, USA), where in case of parameter estimation the first-order conditional estimation method with interaction (FOCE-I) was used. R (version 4.1.3) was used for data processing, model evaluation by visualization of goodness-of-fit (GOF) plots and final uptake predictions.

## Results

### Patients and data

**Fig. 3:**
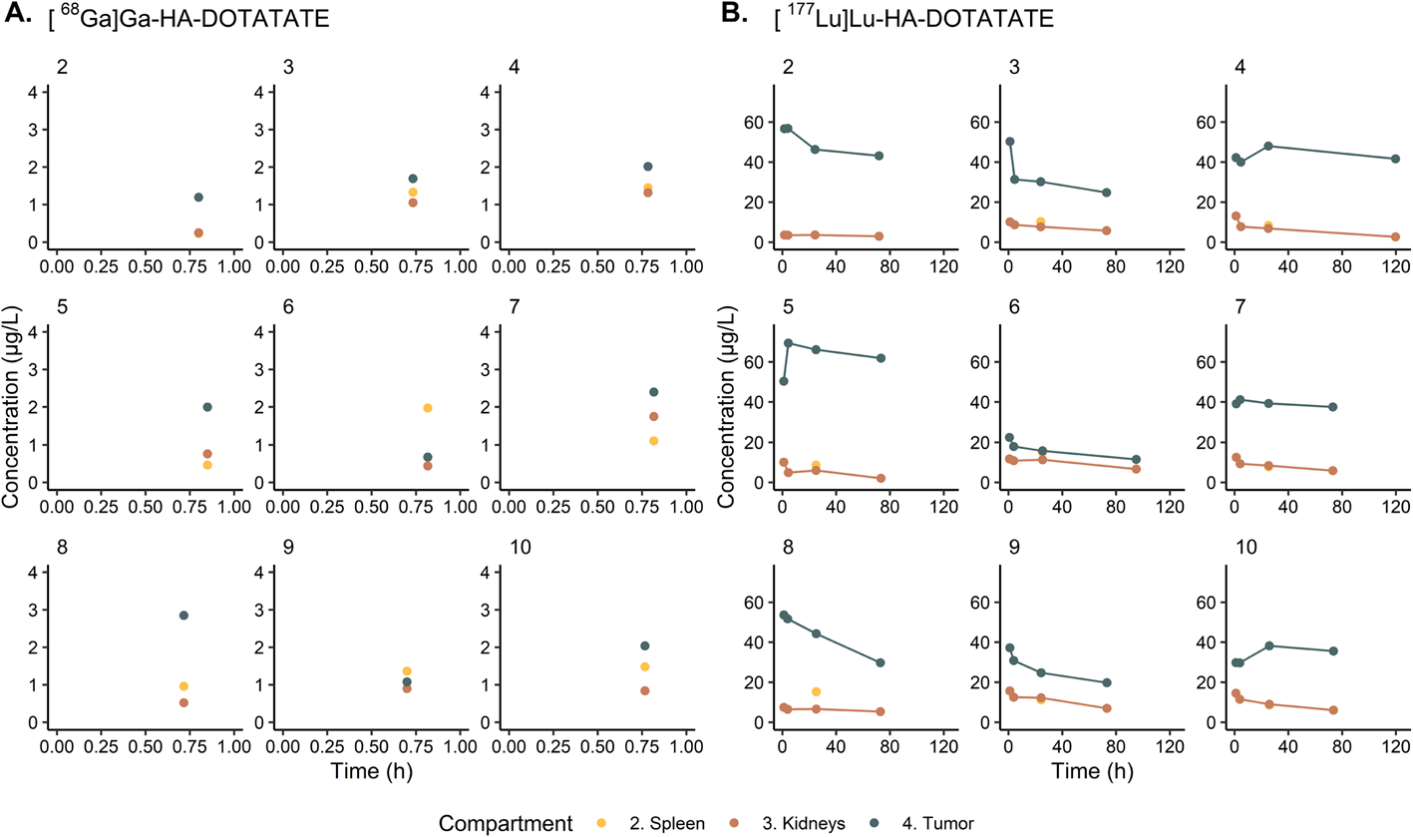
Individually observed concentrations (µg/L) of **A** [^68^Ga]Ga-HA-DOTATATE and **B** [^177^Lu]Lu-HA-DOTATATE in the spleen, kidney and tumors (stratified for patients 2 to 10)

Data from ten patients with NETs who received a diagnostic [^68^Ga]Ga-HA-DOTATATE PET/CT and PRRT with [^177^Lu]Lu-HA-DOTATATE were included. Patient one was excluded, since tumor lesions were not suitable for accurate quantitative analysis as diameters were less than two cm. For patient two, kidney data were excluded from analysis because only one kidney was available for quantitative analysis. Patient characteristics are shown in Table 1. Median (range) administered peptide amounts were 5.23 µg (3.01–9.64 µg) and 151 µg (132–178 µg) for [^68^Ga]Ga-HA-DOTATATE and [^177^Lu]Lu-HA-DOTATATE, respectively. Median (range) tumor volume of target tumors (representing the tumor compartment) was 80.0 mL (7.81–212 mL), while median (range) total tumor volume was 283 mL (22.4–644 mL). Individual [^68^Ga]Ga-HA-DOTATATE and [^177^Lu]Lu-HA-DOTATATE peptide concentration–time curves (based on imaging data) for spleen, kidney and tumor are shown in Fig. 3.

**Table 1:** Patient characteristics

Characteristic	Median (range) or number (%)
*Sex*	
Male (n)	4 (44%)
Female (n)	5 (56%)
Age (years)	70 (44–76)
Weight (kg)	75.0 (55.0–108)
Height (cm)	174 (160–189)
Creatinine clearance (mL/min)	68.8 (53.6–111)
Tumor volume of target tumors (representing the tumor compartment)^a^ (mL)	80.0 (7.81–212)
Total tumor volume^b^ (mL)	283 (22.4–644)
*Injected radioactivity*	
[^68^Ga]Ga-HA-DOTATATE (MBq)	96.0 (75.6–102)
[^177^Lu]Lu-HA-DOTATATE (MBq)	7271 (7176–7613)
*Injected peptide amount*	
[^68^Ga]Ga-HA-DOTATATE (µg)	5.23 (3.01–9.64)
[^177^Lu]Lu-HA-DOTATATE (µg)	151 (132–178)

^a^Target tumors represent lesions with a diameter > 2 cm and a maximum of five segmented lesions of which two per organ system per patient

^b^Sum of tumor volumes of all tumors (including target tumors)

### Semi-physiological PK model

All PK parameters for the [^68^Ga]Ga-HA-DOTATATE and [^177^Lu]Lu-HA-DOTATATE models are shown in Table 2. Population uptake rate parameters (*k*_in_) for spleen, kidney and tumors differed by a 0.29- (15% relative standard error (RSE)), 0.49- (15% RSE) and 1.43-fold (14% RSE), respectively, for [^177^Lu]Lu-HA-DOTATATE compared to [^68^Ga]Ga-HA-DOTATATE. Model fits adequately described observed data in kidney and tumors for both peptides. GOF plots of the [^68^Ga]Ga-HA-DOTATATE and [^177^Lu]Lu-HA-DOTATATE model are shown in Additional file 1: Figures S1 and S2, respectively. In addition, *η* values were normally distributed.

**Table 2:** Overview of final model parameter differences for [^68^Ga]Ga-HA-DOTATATE and [^177^Lu]Lu-HA-DOTATATE, where compartments represent blood (1), spleen (2), kidney (3), tumor (4), a SSTR-expressing (5) and rest (6) compartment

	Parameter values (RSE% provided for estimated values*)	
	[^68^Ga]Ga-HA-DOTATATE	[^177^Lu]Lu-HA-DOTATATE
***Structural parameters***		
*k*_10_ (h^−1^)	0.25	0.575
*k*_12_ (h^−1^)	0.21	0.0607 (0.29-fold difference (15%))
*k*_20_ (h^−1^)	0.01	0.01
*k*_13_ (h^−1^)	0.22	0.107 (0.49-fold difference (15%))
*k*_30_ (h^−1^)	0.01	0.01
*k*_14_ (h^−1^)	0.11	0.157 (1.43-fold difference (14%))
*k*_40_ (h^−1^)	0.01	0.00375 (0.38-fold difference (35%))
*k*_15_ (h^−1^)	2.5	2.5
*k*_50_ (h^−1^)	0.01	0.01
*k*_16_ (h^−1^)	1	1
*k*_60_ (h^−1^)	0.01	0.01
V1 (L)	4	4
V2 (L)	0.21	0.21
V3 (L)	0.3	0.3
V5 (L)	4	4
V6 (L)	50	50
Fraction unbound in plasma	0.69	0.57
B_MAX_ compartment 2 (nmol/L)	16.7	16.7
B_MAX_ compartment 3 (nmol/L)	6.7	6.7
B_MAX_ compartment 4 (nmol/L)	30	30
B_MAX_ compartment 5 (nmol/L)	2.4	2.4
***IIV (CV%)***		
*k*_10_	31.6%	31.6%
*k*_12_ or BMAX compartment 2**	50%	50%
*k*_13_ or BMAX compartment 3**	50%	50%
*k*_14_ or BMAX compartment 4**	50%	50%
*k*_15_ or BMAX compartment 5**	31.6%	31.6%
***Structural effect***		
Tumor volume on k_14_	1	0.67 (17%)
***RUV***		
Proportional error (CV%)	31.6%	31.6%

RSEs were obtained from the NONMEM covariance step. The tumor compartment volume parameter (V4) was based on individually measured tumor volumes

*B*_MAX_, maximum binding capacity; V, compartment volume; CV%, coefficient of variation; IIV, inter-individual variability; RSE, relative standard error; RUV, residual unexplained variability

*PK parameters for [^177^Lu]Lu-HA-DOTATATE were estimated as a fold difference compared to the parameter for [^68^Ga]Ga-HA-DOTATATE

**IIV was added to k parameters for [^68^Ga]Ga-HA-DOTATATE, while for [^177^Lu]Lu-HA-DOTATATE IIV was added to B_MAX_

Maximum receptor occupancy after administration of [^177^Lu]Lu-HA-DOTATATE ranged from 39 to 55% for spleen, 71–97% for kidney and 78–100% for tumors and this occupancy decreased over time for all compartments.

### Individual [^177^Lu]Lu-HA-DOTATATE predictions

Predictions for [^177^Lu]Lu-HA-DOTATATE concentration–time curves based on individual Bayesian PK parameter estimates for [^68^Ga]Ga-HA-DOTATATE PK showed reasonably well individual fits for tumors (RPE for AUC in the range of −40 to 28%). Predicted individual kidney concentration–time curves were less accurate, with RPE for AUC between −53 and 41%. Individual AUC prediction versus observation and RPE results for kidney and tumor are shown in Table 3. For absorbed doses, differences between individual predictions and observations were more pronounced (see Table 4), with RPEs for tumor ranging from −51 to 44%, while for kidney this was −58 to 82%. All individual [^177^Lu]Lu-HA-DOTATATE predictions and observations are presented in Additional file 1: Figure S3.

**Table 3:** Individual tumor and kidney AUC predictions versus observations for [^177^Lu]Lu-HA-DOTATATE, followed by calculated RPEs per patient (ID2–ID10)

AUC_0-tlast_ (mg*h/L)	ID2	ID3	ID4	ID5	ID6	ID7	ID8	ID9	ID10
*Tumor*									
Observed	3.32	2.09	5.29	4.69	1.38	2.80	2.94	1.76	2.59
Predicted	2.95	2.67	4.05	2.89	0.825	3.06	3.16	1.50	2.60
RPE	−11%	28%	−23%	−38%	−40%	9%	8%	−14%	0%
*Kidney*									
Observed	–	0.520	0.634	0.334	0.902	0.564	0.443	0.764	0.621
Predicted	–	0.533	0.680	0.469	0.408	0.479	0.410	0.496	0.426
RPE	–	4%	14%	41%	−53%	−12%	−10%	−32%	−32%

AUC_0-tlast_, area under the curve, calculated from time is 0 to time of the last measurement; RPE, relative prediction error (((predicted – observed)/observed) * 100%)

**Table 4:** Individual tumor and kidney absorbed dose predictions versus observations for [^177^Lu]Lu-HA-DOTATATE, followed by calculated RPEs per patient (ID2–ID10)

Absorbed dose (Gy)	ID2	ID3	ID4	ID5	ID6	ID7	ID8	ID9	ID10
*Tumor*									
Observed	24.4	14.4	29.3	38.3	8.67	34.4	19.5	12.4	27.7
Predicted	21.3	17.4	19.6	18.7	5.62	25.5	28.1	12.1	21.5
RPE	−13%	21%	−33%	−51%	−35%	−26%	44%	−3%	−23%
*Kidney*									
Observed	–	3.12	2.21	1.19	5.38	3.92	3.79	4.41	3.71
Predicted	–	2.57	2.57	2.69	2.17	2.26	2.81	2.51	2.75
RPE	–	33%	−18%	21%	82%	−58%	−28%	−34%	−38%

RPE, relative prediction error (((predicted – observed)/observed) * 100%)

**Fig. 4:**
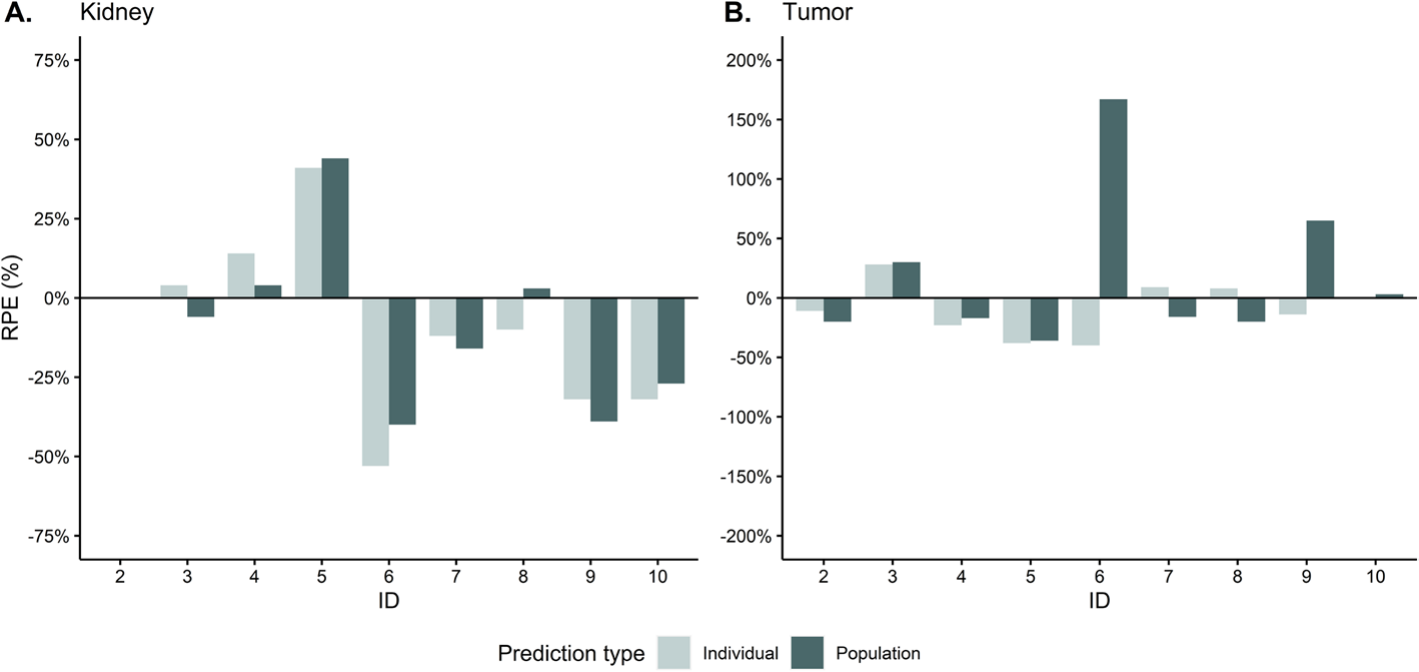
Comparison of individual and population relative prediction errors (RPEs) for kidney (**A**) and tumors (**B**), stratified per individual, based on predicted vs observed area under the curves of [^177^Lu]Lu-HA-DOTATATE

Individual RPEs were compared to RPEs based on the final population model estimates for tumors and kidney AUC predictions (see Fig. 4). For tumors, individual predictions outperformed population predictions in seven of nine cases. However, for kidney the population predictions seemed to perform better for four of eight patients.

## Discussion

A semi-physiological PK model was developed to predict [^177^Lu]Lu-HA-DOTATATE kidney and tumor uptake based on single-time-point pre-therapeutic PET/CT imaging with [^68^Ga]Ga-HA-DOTATATE, to pave the way for image-based treatment planning in PRRT. This physiologically informed population PK model provided insights in population trends and allowed individual Bayesian estimation of absorbed doses. This is an advantage compared to conventional PBPK models, where a bottom-up approach is used and variability in individual differences is not identified but needs to be included beforehand (on many different parameters) to describe variability in the data [20]. Moreover, all relevant physiological parameters that could impact radiopharmaceutical distribution or uptake were still included in the model, and the simpler model structure (compared to full PBPK models) enabled straightforward comparison of both radiopharmaceuticals. This provided a first prediction of the PK profiles of both radiopharmaceuticals in NET patients, taking into account relevant system- and drug-specific parameters as well as IIV and structural effects.

### Semi-physiological model development

The tumor sink effect (i.e., reduced organ uptake with increasing total tumor volume) was assumed similar for [^68^Ga]Ga-HA-DOTATATE and [^177^Lu]Lu-HA-DOTATATE. However, a more profound sink effect due to structural difference might be hypothesized during therapy, since the current study showed that biodistribution of both agents was also different (i.e., [^177^Lu]Lu-HA-DOTATATE showed a lower spleen and kidney uptake, while tumor uptake rates were higher compared to [^68^Ga]Ga-HA-DOTATATE). Still, the assumption of the effect being similar (i.e., the effect for an individual being within the same order of magnitude for both agents) seemed most suitable, since we do not have any information based on population data regarding a tumor sink effect during PRRT. For [^177^Lu]Lu-HA-DOTATATE, the degradation rate in tumors (*k*_40_) was estimated based on data, which resulted in a lower rate (0.00375 h^−1^) compared to rate constant used for [^68^Ga]Ga-HA-DOTATATE (0.01 h^−1^). This degradation rate in tumor being different does not per se reflect a difference in degradation between both radiopharmaceuticals, but was the result of the fact that this rate constant for [^68^Ga]Ga-HA-DOTATATE could not be estimated based on a single observation per individual.

### Relevance of PK differences

Unfortunately, data regarding radionuclide therapies is often limited with sparse data observations. Still, our approach using a lumped semi-physiological PK model (based on a previously developed PBPK model and literature data) proved that these limited data are still useful for model development (based on fixed parameters) (see Additional file 1: Figures S1 and S2). Model results showed clear differences in uptake rate parameters for spleen, kidney and tumors for [^68^Ga]Ga-HA-DOTATATE compared to [^177^Lu]Lu-HA-DOTATATE. The reduced uptake rate in kidney and spleen for [^177^Lu]Lu-HA-DOTATATE was accompanied by increased uptake into the tumor compartment, which was also reflected by an increased uptake rate for tumors (*k*_14_). For kidneys, a lower uptake rate was to be expected, since [^177^Lu]Lu-HA-DOTATATE infusion is co-administered with an amino acid solution for renal toxicity prevention. This solution results in less proximal reabsorption and thus kidney uptake is expected to be reduced compared to [^68^Ga]Ga-HA-DOTATATE [40]. These relevant differences in PK parameters, and thus mismatches in distribution profiles between both radiopharmaceuticals, reflect clinical observations and have also previously been reported in literature [5–10].

### Individual [^177^Lu]Lu-HA-DOTATATE predictions

Our semi-physiological PK model showed that [^177^Lu]Lu-HA-DOTATATE tumor AUCs could be predicted reasonably well based on individual [^68^Ga]Ga-HA-DOTATATE scan data (with tumor AUC RPEs ranging between −40 and 28%). In addition, for tumors, individual predictions based on Bayesian parameter estimates for [^68^Ga]Ga-HA-DOTATATE outperformed population predictions for [^177^Lu]Lu-HA-DOTATATE for seven out of nine patients, confirming that diagnostic imaging provides useful information for [^177^Lu]Lu-HA-DOTATATE uptake. For the two patients where RPEs were lower for population predictions, individual uptake predictions were still accurate and differences between the individual and population approach were very small. For patient six, a clear benefit for using diagnostic imaging was shown, since the RPE based on the population model was extremely high for the tumor prediction, whereas the individual prediction (informed by diagnostic imaging) resulted in a smaller RPE and thus improved prediction (see Fig. 4). Still, this patient showed highest RPEs for both kidney and tumor, which could not be explained after looking at further patient details (such as patient characteristics, renal function and renogram scan, number and sites of metastasis, etc.). All in all, results showed that inter-individual differences in PK parameters for tumor uptake on [^68^Ga]Ga-HA-DOTATATE were informative for expected PRRT uptake. However, for kidney AUC predictions were less accurate (RPEs −53 to 41%), and even more so for absorbed doses (RPEs −58 to 82%). This could be due to the complexity of interpretation of kidney scan observations. Observed radioactivity in kidneys does not only reflect intracellular uptake, but also radioactivity in urine due to renal excretion. Especially for [^68^Ga]Ga-HA-DOTATATE, with scan-acquisition times shortly after administration, the contribution of activity in urine is probably high. This might lead to inaccurate individual estimates of the kidney uptake parameter (*k*_13_), which on its turn will result in less accurate parameter estimates for [^177^Lu]Lu-HA-DOTATATE and thus less adequate uptake predictions. In addition, diagnostic imaging scan protocols are less stringent compared to PRRT protocols, for example, regarding supervision of patients’ hydration status and co-administration of amino acid solutions. These protocol aspects could result in higher variability between patients for [^68^Ga]Ga-HA-DOTATATE compared to PRRT with [^177^Lu]Lu-HA-DOTATATE and these factors might contribute to the remaining mismatches in prediction of PRRT accumulation based on diagnostic imaging. Another factor that might cause uncertainty in our predictions is that population organ volumes rather than individually measured organ volumes were used. However, we assume this did not substantially impact individual model predictions. After all, we do not expect the model parameters to change with different organ volumes and potential individual differences are already taken into account in predictions by using an intra-patient prediction approach.

In general, various known and unknown external factors can influence visualization and quantification of radiopharmaceutical distributions. The physical characteristics of radionuclides and the acquisition techniques themselves may also result in (structural) differences between ^68^Ga and ^177^Lu imaging. For instance, noise levels in the imaging data, voxel sizes and the methods applied for quantification vary greatly between ^68^Ga-PET/CT and ^177^Lu-SPECT/CT, which give rise to variations in quantification of activity concentrations. Details regarding the uncertainty in image acquisition and post-processing are beyond the scope of this article, but more information can be found in previously published articles [41–43].

Results for AUC (µg*h/L) and absorbed dose (Gy) clearly differed for some patients, by means that AUC RPE was accurate and absorbed dose RPE was not, or vice versa. This inconsistency was caused by the different time intervals that were used in calculations of absorbed dose compared to AUC. For AUCs, integrated concentrations over time were determined up to time of the last measurement, while for absorbed dose, the cumulative radioactivity was extrapolated up to infinity based on individual elimination rate constants. This highlights the uncertainty in dosimetry methods, where extrapolations to later time points highly impact absorbed dose calculations.

### Clinical implications

A method was developed to individually predict [^177^Lu]Lu-HA-DOTATATE PK based on single-time-point imaging with [^68^Ga]Ga-HA-DOTATATE. Individual tumor predictions appeared less complex compared to kidney and, for tumor, it was shown that addition of information derived from diagnostic imaging improved the individual uptake predictions. However, apart from evident differences in PK parameters, there still are other factors that affect distribution differences between [^68^Ga]Ga-HA-DOTATATE and [^177^Lu]Lu-HA-DOTATATE.

The developed model based on data from only nine patients provides a framework for precision medicine of PRRT. Using this approach, tumor AUCs were predicted reasonably well with a RPE of approximately ± 30%. The question remains what prediction error is acceptable for applying this approach for individualized dosing in clinical care. Of course, with nuclear imaging, day-to-day or inter-scan variability exists, which reflects a general intra-patient variability in accumulation that is observed in case nuclear imaging is repeated. We assumed this to be in the order of ~15 to 20% for [^68^Ga]Ga-HA-DOTATATE PET/CT (based on clinical observations as well as results from PSMA-PET/CT imaging [44]). However, to determine maximal ranges for prediction errors, additional knowledge is warranted on variability in uptake in this population as well as the effect of tumor and kidney accumulation on efficacy and toxicity outcomes, respectively, and whether, for example, a 20% increased or decreased uptake will impact these outcomes. Possibly, prediction error requirements might even be different for kidneys (organ at risk) compared to tumors (target tissue). Once such requirements are determined, this modeling approach could help to guide individualized dosing based on expected uptake and individual deviation from the typical population. Another, somewhat similar, approach could be to individualize dosing guided based on uptake in the first PRRT cycle instead of the diagnostic imaging.

## Conclusion

Semi-physiological population PK models were developed for [^68^Ga]Ga-HA-DOTATATE and [^177^Lu]Lu-HA-DOTATATE. PK parameter differences showed lower uptake rate parameters for kidney and spleen (0.29- and 0.49-fold, respectively), while the tumor uptake rate was 1.43-fold higher for [^177^Lu]Lu-HA-DOTATATE compared to [^68^Ga]Ga-HA-DOTATATE. Based on these developed models, individual Bayesian estimates for [^68^Ga]Ga-HA-DOTATATE were used to calculate individual PK parameters for [^177^Lu]Lu-HA-DOTATATE and subsequently predict [^177^Lu]Lu-HA-DOTATATE uptake into different compartments. Although tumor AUC predictions were more accurate compared to kidney (range in relative prediction errors −40 to 28% and −53 to 41%, respectively), absorbed dose predictions were less adequate for both compartments. Many aspects, additional to PK differences, play a part in the challenging translation of [^68^Ga]Ga-HA-DOTATATE to [^177^Lu]Lu-HA-DOTATATE distribution, but this framework already confirmed that diagnostic imaging provides useful information to predict [^177^Lu]Lu-HA-DOTATATE tumor uptake.

### Supplementary Information

**Additional file 1**: **Figure S1**. Goodness-of-fit plots for [^68^Ga]Ga-HA-DOTATATE based on the final semi-physiological population PK models. **Figure S2**. Goodness-of-fit plots for [^177^Lu]Lu-HA-DOTATATE based on the final semi-physiological population PK models. **Figure S3**. Individual tumor and kidney predictions and observations for [^177^Lu]Lu-HA-DOTATATE based on individual PK parameters [^68^Ga]Ga-HA-DOTATATE using the final semi-physiological population PK models.

## Data Availability

The datasets used for the current study are available from the corresponding author on reasonable request.

## Abbreviations

^177^Lu: Lutetium-177
^68^Ga: Gallium-68
AUC: Area under the curve
*B*_MAX_: Maximal binding capacity
CV: Coefficient of variation
GOF: Goodness-of-fit
ICRP: International commission on radiological protection
IIV: Inter-individual variability
NET: Neuroendocrine tumor
NLMEM: Nonlinear mixed-effects model
PBPK: Physiologically based pharmacokinetic
PK: Pharmacokinetic(s)
PRRT: Peptide receptor radionuclide therapy
RPE: Relative prediction error
RSE: Relative standard error
RUV: Residual unexplained variability
SSA: Somatostatin analogue
SSTR: Somatostatin receptor
TAC: Time–activity curve
V: Compartment volume
