# Supplementary material for: Predicting [177Lu]Lu-HA-DOTATATE kidney and tumor accumulation based on [68Ga]Ga-HA-DOTATATE diagnostic imaging using semi-physiological population pharmacokinetic modeling
Source: EJNMMI Phys. 2023 Aug 24;10:48. doi: 10.1186/s40658-023-00565-4 (PMC10449733; doi:10.1186/s40658-023-00565-4)
Supplement: Supplementary file 1 — Additional file 1: Figure S1. Goodness-of-fit plots for [68Ga]Ga-HA-DOTATATE based on the final semi-physiological population PK models. Figure S2. Goodness-of-fit plots for [177Lu]Lu-HA-DOTATATE based on the final semi-physiological population PK models. Figure S3. Individual tumor and kidney predictions and observations for [177Lu]Lu-HA-DOTATATE based on individual PK parameters [68Ga]Ga-HA-DOTATATE using the final semi-physiological population PK models. [file 40658_2023_565_MOESM1_ESM.docx]

**Supplementary Materials**

**Predicting [^177^Lu]Lu-HA-DOTATATE kidney and tumor accumulation based on [^68^Ga]Ga-HA-DOTATATE diagnostic imaging using semi-physiological population pharmacokinetic modelling**

Hinke Siebinga^1,2^, Berlinda J. de Wit-van der Veen^2^, Jos H. Beijnen^1^, Marcel P.M. Stokkel^2^, Thomas P.C. Dorlo^1,3^, Alwin D.R. Huitema^1,4,5^, Jeroen J.M.A. Hendrikx^1,2^

1 Department of Pharmacy & Pharmacology, The Netherlands Cancer Institute, Amsterdam, The Netherlands

2 Department of Nuclear Medicine, The Netherlands Cancer Institute, Amsterdam, The Netherlands

3 Department of Pharmacy, Uppsala University, Uppsala, Sweden

4 Department of Clinical Pharmacy, University Medical Center Utrecht, Utrecht University, Utrecht, The Netherlands

5 Department of Pharmacology, Princess Máxima Center for Pediatric Oncology, Utrecht, The Netherlands

**Corresponding author:** h.siebinga@nki.nl, Plesmanlaan 121, 1066 CX Amsterdam

**First author:** h.siebinga@nki.nl, Plesmanlaan 121, 1066 CX Amsterdam

**Figure S1 – Goodness-of-fit plots for [^68^Ga]Ga-HA-DOTATATE based on the final semi-physiological population PK models.**

**
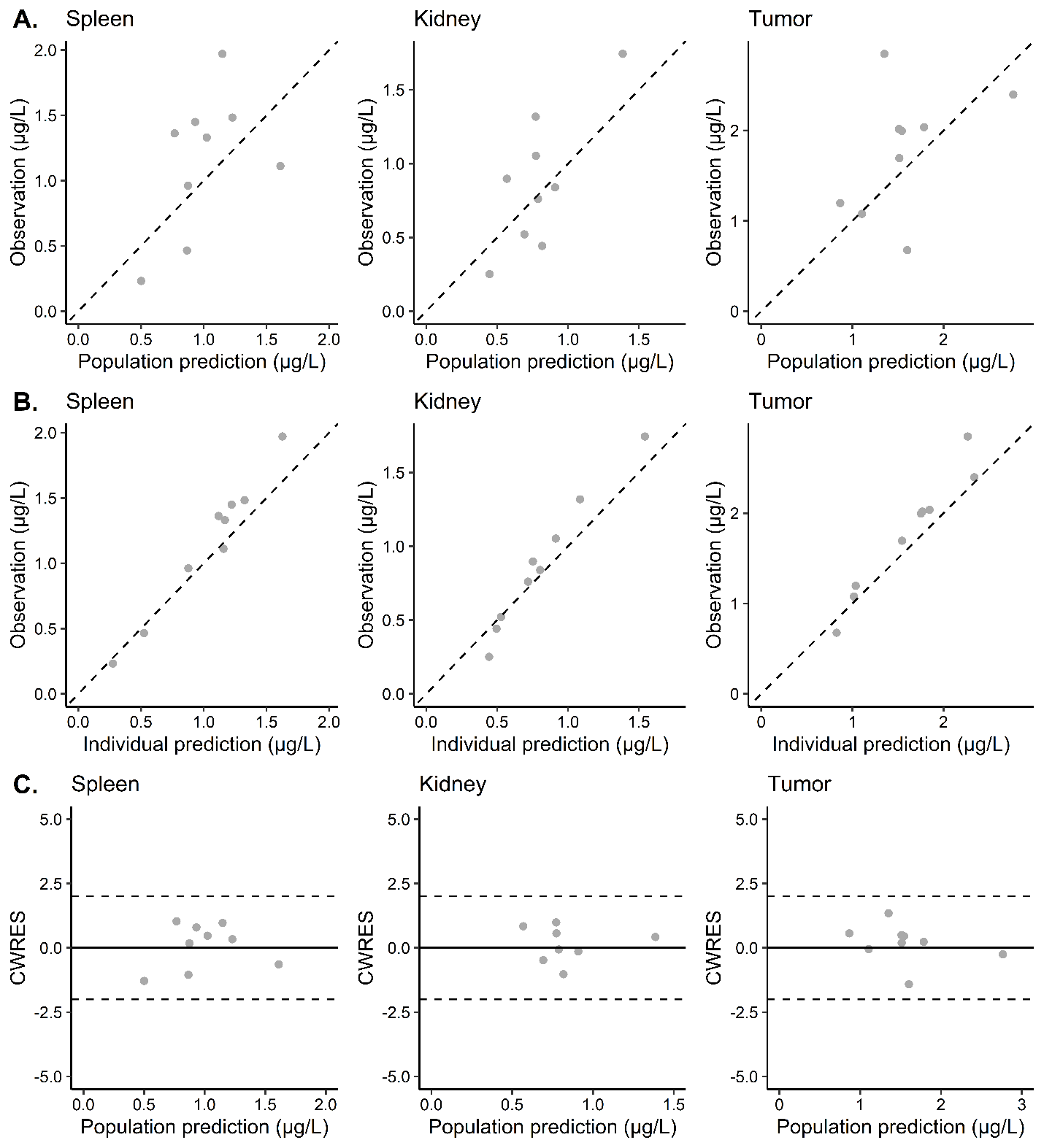
**

**Figure S2 – Goodness-of-fit plots for [^177^Lu]Lu-HA-DOTATATE based on the final semi-physiological population PK models.**

**
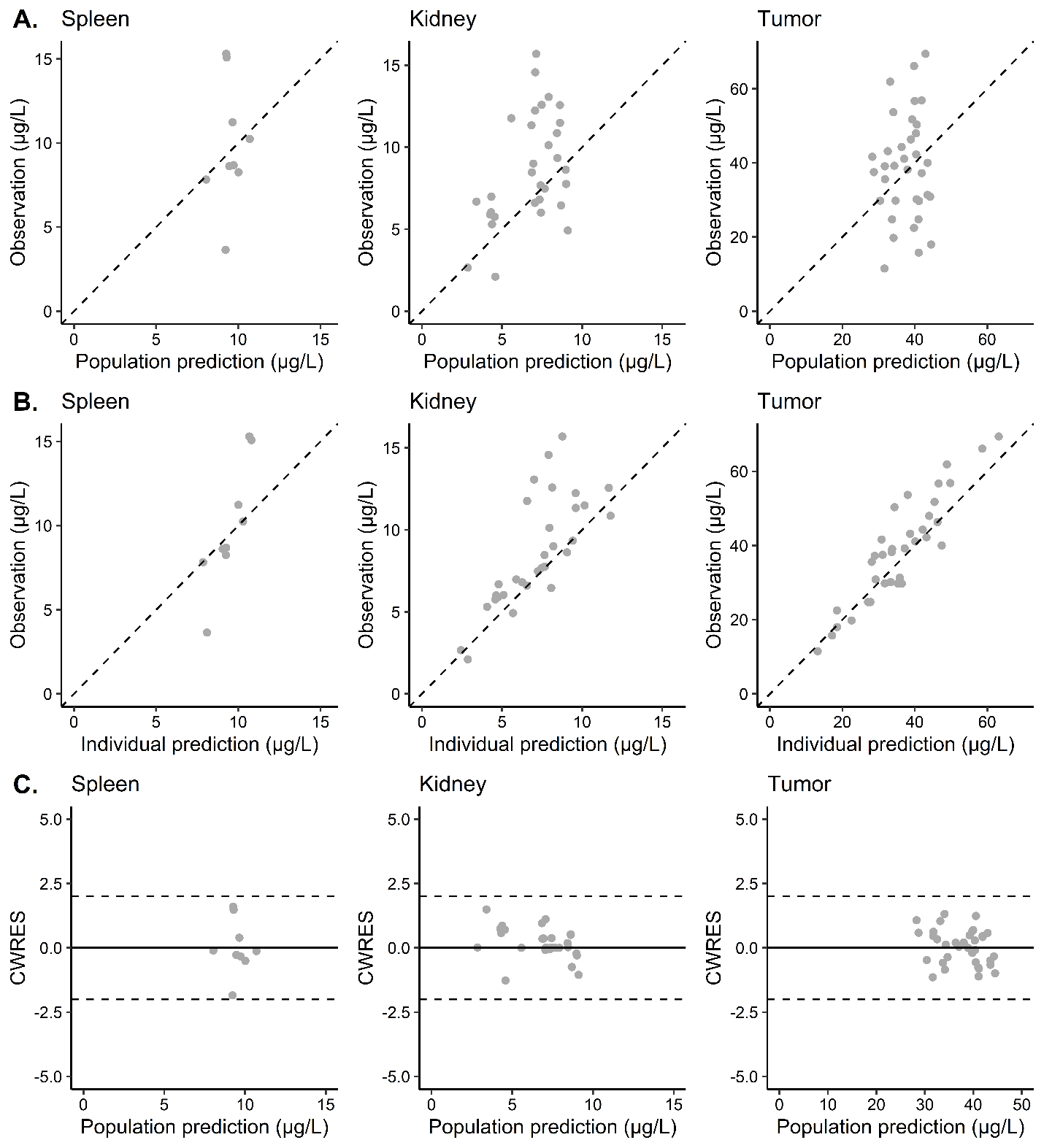
**

**Figure S3 – Individual tumor and kidney predictions and observations for [^177^Lu]Lu-HA-DOTATATE based on individual PK parameters [^68^Ga]Ga-HA-DOTATATE using the final semi-physiological population PK models.**

**
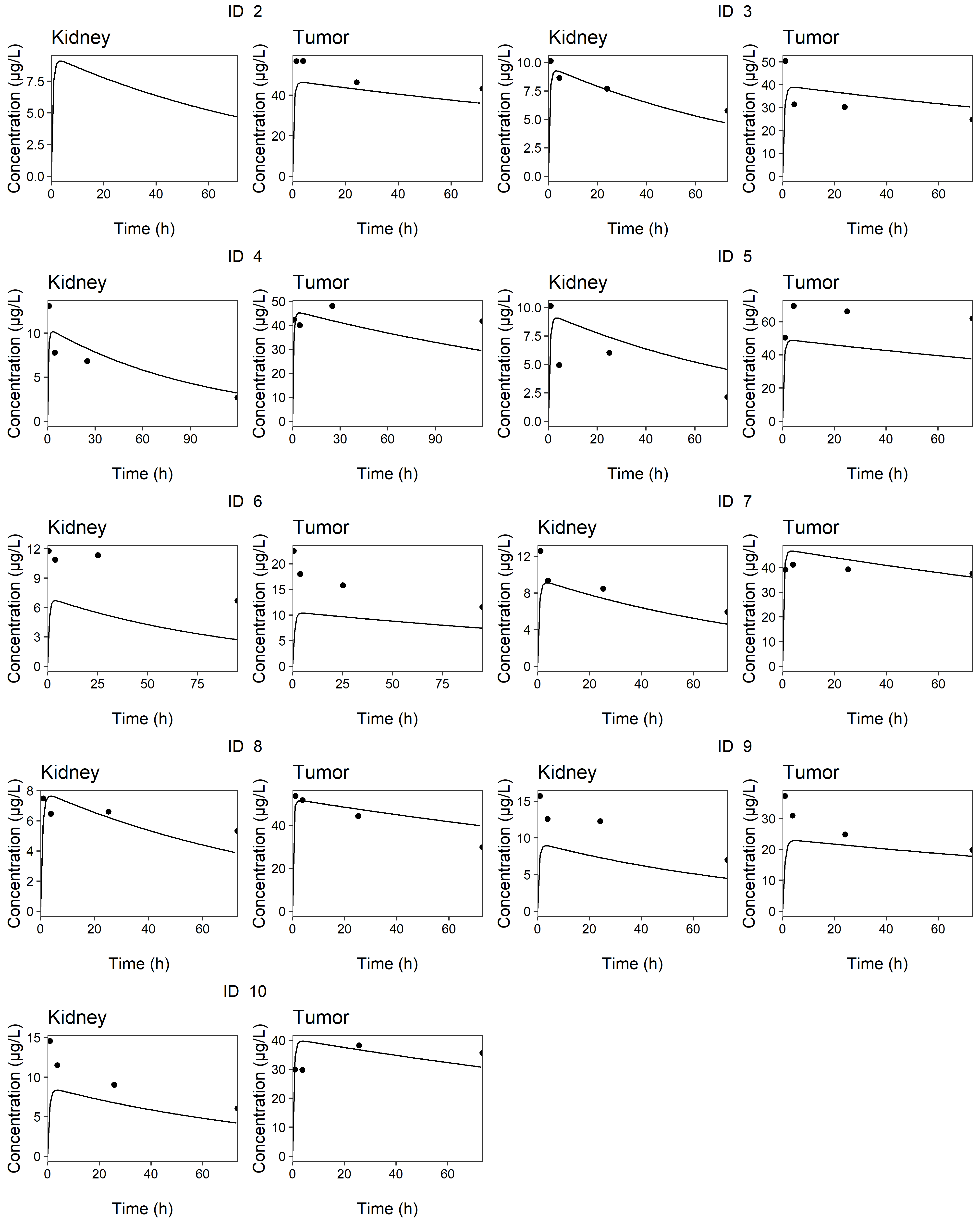
**
